# Associations Between Hearing Loss and Depressive Symptom Trajectories in Middle-Aged and Older People in China: Retrospective Analysis

**DOI:** 10.2196/75545

**Published:** 2025-11-24

**Authors:** Yuchen Liu, Wenwen Liu, Jun Ma, Yangfan Chai, Guilan Kong

**Affiliations:** 1 National Institute of Health Data Science Peking University Beijing China; 2 Peking University Third Hospital Beijing China; 3 Peking University Chongqing Research Institute of Big Data Chongqing China; 4 Advanced Institute of Information Technology Peking University Hangzhou, Zhejiang China

**Keywords:** depression symptom, latent growth mixture model, trajectory analysis, China Health and Retirement Longitudinal Study, CHARLS

## Abstract

**Background:**

Hearing loss and depression are important health issues among the middle-aged and older population.

**Objective:**

This study aimed to investigate the associations between hearing loss and depressive symptom trajectories in the Chinese middle-aged and older adult population.

**Methods:**

The survey data of 2011, 2013, 2015, and 2018 waves collected in the China Health and Retirement Longitudinal Study were used for analysis. The latent growth mixture modeling approach was used to explore the trajectories of depressive symptoms. Hearing loss was identified through self-reporting, and depressive symptoms were assessed using the 10-item Center for Epidemiologic Studies Depression scale. The associations between hearing loss and depressive symptom trajectories were examined using logistic regression models.

**Results:**

A total of 4768 participants without depressive symptoms at baseline were included for analysis. Among them, 4 depressive symptom trajectories, including “stable low symptoms” (n=3656, 76.68%), “slowly progressing symptoms” (n=503, 10.55%), “relieved symptoms after progression” (n=467, 9.79%), and “rapidly progressing symptoms” (n=142, 2.98%) were identified. Hearing loss was found to be significantly associated with the trajectory of “rapidly progressing symptoms.”

**Conclusions:**

The trajectories of depressive symptoms in middle-aged and older people have 4 types with distinct patterns. Hearing loss is associated with the progression of depressive symptoms, and its impact is more significant for males, affecting not only symptom severity but also progression speed. These findings indicate that the mental health status of middle-aged and older people with hearing loss requires careful consideration, and timely interventions should be provided.

## Introduction

Depression is a common mental illness that may seriously damage human health, and it is usually caused by the complex interactions between social, psychological, and biological factors. Various life difficulties, such as childhood trauma, loss of loved ones, unemployment, and chronic diseases such as arthritis, asthma, cardiovascular disease, and cancer, can lead to or exacerbate depressive symptoms. The World Health Organization [1] reported that approximately 5% of adults worldwide experience depressive disorders. The consequences of depression may affect every aspect of life, cause significant mental distress, and, in some cases, even lead to suicide. According to the 2017 Global Burden of Diseases study [2], depression now ranks as the third leading cause of years lived with disability. An epidemiological study [3] reported that the lifetime prevalence of depression in Chinese adults was 8% for female individuals and 5.7% for male individuals, and the 12-month depression prevalence was 4.2% for female individuals and 3% for male individuals. Among individuals aged 50 years and above, lifetime depression prevalence ranged from 7.3% to 7.8%, and 12-month depression prevalence ranged from 3.8% to 4.1%.

Most existing studies on depression [4-6] used the total score on an assessment scale as a quantitative measure to determine the severity level of depression or depressive symptoms of an individual, and this approach treats the population as a homogeneous group with a common pattern of depressive symptom progression. However, some studies have found heterogeneity in depressive symptoms among different groups, which necessitates individual-centered research methods to tailor interventions. Among the existing individual-centered studies on depression, the majority are cross-sectional and use the severity of depressive symptoms or specific symptoms as features to categorize patients [7-9]. In practice, the longitudinal patterns of depressive symptoms can reflect the heterogeneity of depression more comprehensively and objectively than the patterns identified using cross-sectional data, and the trajectories of depressive symptoms have gradually attracted research attention in recent years [10-12].

To explore the trajectories of or changes in depressive symptoms over time, latent growth modeling approaches have been used to identify unobserved latent groups within different study populations [12-14]. Latent growth modeling approaches consider individuals to be part of a heterogeneous population consisting of unobserved groups with similar development patterns [13,15]. One of the primary methods for latent growth modeling is latent growth mixture modeling (LGMM) [16], which enables the modeling of within-class heterogeneity [14], thereby facilitating a more accurate identification of the underlying trajectories of disease progression among different individuals. For example, Maccallum et al [16] used the LGMM method to portray depression trajectories following spousal and child bereavement using the Health and Retirement Study, resulting in the identification of 4 distinct types of depression: resilience (little or no depression; 68.2%), chronic grief (an onset of depression following loss; 13.2%), depressed-improved (high preloss depression that decreased following loss; 11.2%), and preexisting chronic depression (high depression at all assessments; 7.4%). Hong et al [17] used LGMM to analyze perinatal depression in Chinese perinatal women based on data from the First Affiliated Hospital of Wenzhou Medical University and identified 2 types of perinatal depressive symptom trajectory, including “decreasing” (95.3%) and “increasing” (4.7%). However, studies about the trajectories of depressive symptoms among middle-aged and older individuals in China are limited.

Hearing, a vital sensory system in humans, is crucial in daily life. Hearing loss profoundly impacts both physical and mental well-being, leading to communication challenges, cognitive decline, limited social interactions, social isolation, financial stress, and a significant reduction in quality of life [18-20]. China’s second national disability survey, conducted in 2006 [21], reported a prevalence of hearing impairment of 2.11%. Hearing loss increases with age, reaching 11.04% among those aged 60 years and above. In the stress process framework, hearing loss is considered a chronic strain, resulting in ongoing, long-term difficulties that negatively impact health and well-being [20]. It may give rise to the accumulation of additional stressors, including stigma and discrimination; poor mental health; and low levels of educational attainment, income, and employment [22].

The associations between hearing loss and depressive symptoms have been explored in some studies. Lee and Hong [23] found that hearing loss significantly contributed to higher rates of depression among solitary females in South Korea. Blay et al [24] investigated the factors of depression among community-dwelling individuals aged 60 years and above in Brazil and observed a significant correlation between hearing loss and depression. A multicenter study of adults aged 50 years or more in the United States also found that among those with hearing loss, the 10-item Center for Epidemiologic Studies Depression (CES-D10) scale score increased by 0.62 points (95% CI 0.23-1.01) for every 10 dB decrease in hearing [25]. Brewster et al [10] also found that age-related hearing loss is associated with increased depressive symptoms in older adults. Another study explored the associations between hearing loss and depressive symptoms in married couples and found that for women, their hearing loss was associated with increased depressive symptoms [26]. Some studies have reported different findings. For example, a study involving 2890 Norwegians aged 60 years and above found a significant association between hearing loss and depression in the baseline cross-sectional data; however, the longitudinal association was not significant over the 6-year follow-up period [27].

Hearing loss may lead to communication difficulties, which can subsequently result in emotional isolation and reduced social participation [28]. In fact, diminished social engagement is a risk factor for depression [29], and recent research shows that about 9% of the association between poor hearing and depressive symptoms could be explained by social isolation. Therefore, social isolation may play a mediating role in the relationship between hearing loss and depression [30]. Some other studies also suggest that there may be neuropsychological mechanisms underlying the association between hearing loss and depressive symptoms [31,32].

As the studies on the longitudinal relationship between hearing loss and depressive symptoms are limited in the literature, we aimed to investigate the depressive symptom trajectories in the middle-aged and older Chinese population first, with a nationally representative large-scale dataset in this study. Furthermore, we explored the relationship between hearing loss and the depressive symptom trajectories. Additionally, we investigated the potential mediating role of social engagement in the relationship between hearing loss and the trajectories of depressive symptoms.

## Methods

### Data Source and Study Population

The China Health and Retirement Longitudinal Study (CHARLS) [33], a nationally representative longitudinal survey of the middle-aged and older population in China, served as the data source. In the CHARLS, information was collected from participants through face-to-face interviews on demographics, physical and mental health status, health-related behaviors and lifestyles, household economic situation, and health insurance. To ensure the representativeness of the samples, the CHARLS used a multistage probability sampling technique to select eligible samples, considering regional and socioeconomic differences. Detailed descriptions of the sampling methods can be found in previous publications [33,34].

**Figure 1:**
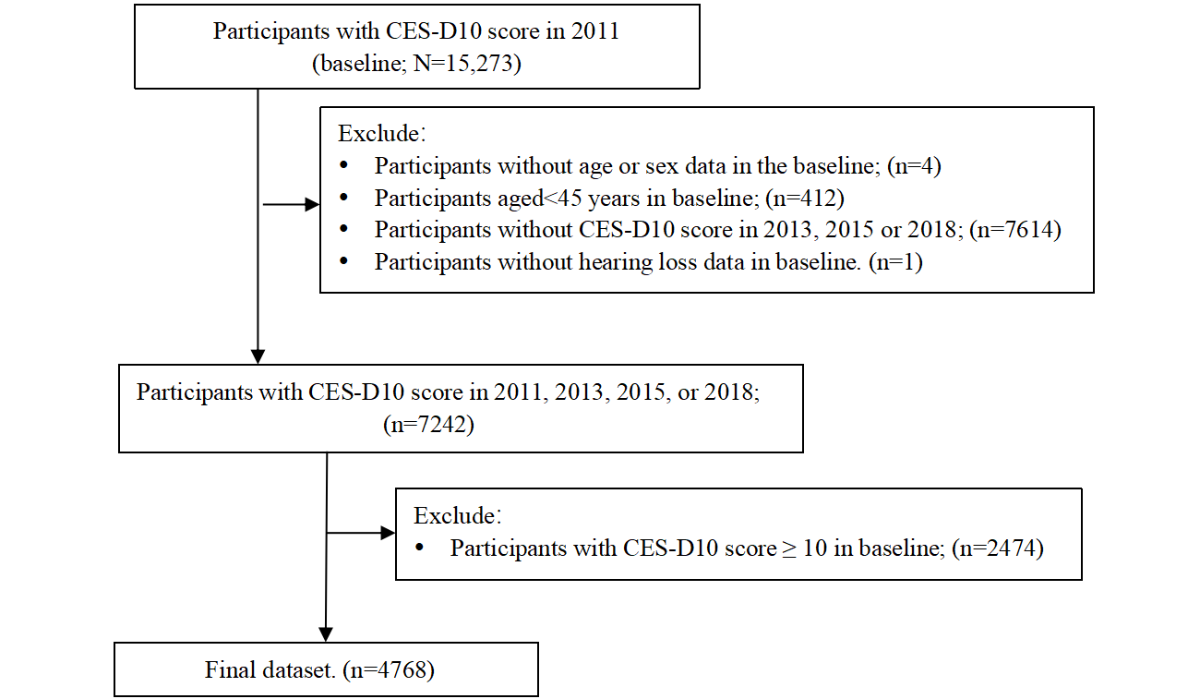
Flowchart of the participant selection. CES-D10: 10-item Center for Epidemiologic Studies Depression.

In total, 15,273 participants involved in the 2011 survey have completed the CES-D10 scale. We then excluded 4 participants without gender or age data; 412 participants with a baseline age below 45 years; 7614 participants who did not participate in the follow-up survey in 2013, 2015, or 2018; and 1 person who had no data on hearing at baseline, resulting in 7242 participants as the potential study population. Furthermore, 2474 participants with depressive symptoms (a CES-D10 score of ≥10) in 2011 were excluded, and ultimately, 4768 participants with complete information were included in the analysis. The participant selection process is shown in Figure 1.

### Measures

#### Depressive Symptoms

The CES-D, developed by Radloff [35], is one of the most commonly used tools for assessing the severity levels of depressive symptoms. It consists of 20 questions, but the response rate is often low due to the large number of questions and the long time needed to answer them. Andresen et al [36] simplified the CES-D and proposed a 10-question version, known as CES-D10, which has been demonstrated to have good reliability and validity in the Chinese population [37].

The depressive symptoms of participants in the CHARLS were measured using the CES-D10, and the detailed questions in the scale are shown in Multimedia Appendix 1. Participants were asked about how they felt and behaved during the last week before the interview, and the response for each question can be selected from the following four choices: (1) very little or not at all (<1 day); (2) not too much (1-2 days); (3) sometimes, or about half the time (3-4 days); and (4) most of the time (5-7 days).

In this study, participants’ answers to the negative questions ranged from “seldom or not at all” to “most of the time” on a scale of 0 to 3. For the positive questions, responses ranging from “rarely or not at all” to “most of the time” were scored from 3 to 0. The total score was calculated by summing the scores of all questions, which ranged from 0 to 30, with higher scores indicating more severe depressive symptoms. According to previous studies [38], we defined a total CES-D10 score of 10 or greater as indicative of depressive symptoms in this study.

#### Hearing Loss

In this study, hearing loss was defined through participants’ self-reports. The CHARLS questionnaire related to hearing is presented in Multimedia Appendix 2, and hearing loss was defined as self-reported hearing loss or partial deafness, typically characterized by the use of a hearing aid or a poor hearing status.

#### Social Engagement

In the CHARLS, information on social engagement was primarily collected through the question “Have you participated in the following social activities in the past month?” with 12 response options: interacted with a friend; played ma-jong, chess, or cards; went to a community club; helped family, friends, or neighbors who do not live with you and did not pay you for the help; went to a sport, social, or other kind of club; took part in a community-related organization; participated in voluntary or charity work; cared for a sick or disabled adult who does not live with you and who did not pay you for the help; attended an educational or training course; invested in stocks; used the internet; other; and none of these. The first 10 activities were selected for analysis. Each activity was coded as 1 (participated) or 0 (did not participate), and the variable of “social engagement” was the total of these 10 items [38].

#### Confounding Variables

Confounding variables in this study included age, gender, education level, area of residence, marital status, smoking status, drinking status, household income, BMI, and comorbidities, including hypertension, diabetes, and cardiovascular disease (CVD). These confounding variables were selected by referring to the factors associated with depressive symptoms reported in previous studies [23,24]. Among these variables, demographics (age, gender, education level, area of residence, marital status, and household income) and health-related behaviors (smoking status and drinking status) were obtained from the questionnaire. BMI was defined as weight (kg) divided by the square of height (m^2^), while weight and height were acquired through physical examination. It was categorized into 4 groups: underweight (<18.5 kg/m^2^), normal (18.5-23.9 kg/m^2^), overweight (24.0-27.9 kg/m^2^), and obese (≥28.0 kg/m^2^) according to the criteria set by the Working Group on Obesity in China [39]. Hypertension was defined as a mean systolic blood pressure of ≥140 mm Hg, a mean diastolic blood pressure of ≥90 mm Hg or more, or self-reported hypertension. Diabetes was defined as a fasting plasma glucose level of ≥126 mg/dL, HbA_lc_ concentration of ≥6.5%, or self-reported having physician-diagnosed diabetes. CVD was defined by self-reported stroke, heart attack, coronary heart disease, angina pectoris, congestive heart failure, or other physician-diagnosed heart problems.

### Statistical Analysis

The baseline characteristics were analyzed using descriptive statistics, with frequencies and percentages used for categorical variables, the χ^2^ test for between-group comparisons, and means (SDs) for continuous variables. Variables included for baseline analysis included age (45-54, 55-64, and ≥65 years); gender (male or female); education level (below high school, high school, or above high school); area of residence (urban or rural); marital status (married and living with a spouse, married and not living with a spouse, separated or divorced, widowed, or unmarried); household income (quintiles 1-5, bottom 20%, 21% to 40%, 41% to 60%, 61% to 80%, and 81% to 100% of income from low to high); smoking status (never smoked, currently smoking, or quit smoking); drinking status (never drank, currently drinking, or quit drinking); BMI (underweight, normal, overweight, or obese); high blood pressure (yes or no); diabetes (yes or no); and CVD (yes or no).

We used the LGMM approach to explore the depressive symptom trajectories of the study population. The model fitting process began with the initial assumption that only one trajectory type existed in the studied population. Subsequently, the number of trajectory types was set to increase gradually until the best-fitting model was found. The performance metrics, including Akaike information criterion, Bayesian information criterion, and sample size–adjusted Bayesian information criterion, were used to identify the best-fitting trajectory model. The smaller the metric values, the better the model is fitted [40]. Some other criteria commonly used for model selection, including a minimum sample size of at least 1% of the total sample for each trajectory category, higher entropy (<0.8), and statistically significant Lo-Mendell-Rubin Likelihood Ratio Test and Bootstrap Likelihood Ratio Test results (*P*<.001), were also considered [41]. Furthermore, the best-fitting model determined the number of trajectory types.

The associations between hearing loss and depressive symptom trajectories were analyzed using logistic regression, with the abovementioned confounding variables as covariates. In logistic regression modeling, we used any pair among the 4 trajectory patterns to form the binary dependent variable, and thus, 6 regression models were constructed for the association analysis. The odds ratios and 95% CIs generated in the regression analysis were reported. We also analyzed the association between hearing loss and social engagement using logistic regression. Furthermore, mediation analysis was conducted to assess the proportion of the association that was mediated through social engagement.

All the data preprocessing, descriptive statistical analysis, and logistic regression analysis in this study were done using STATA MP17. The “sgmediation” package in STATA was used to perform mediation analysis, and the LGMM analysis was done using Mplus 8.7.

### Ethical Considerations

Our study used publicly available anonymized data from the CHARLS and did not require further review. The CHARLS was approved by the Biomedical Ethics Review Committee of Peking University (IRB00001052-11015). The original ethical approval and participant consent included the use of data, and all participants signed an informed consent form before participation.

## Results

A total of 4768 participants, with a mean age of 56.42 (SD 7.87) years, were included in the final analysis. The baseline characteristics of the included participants are shown in Table 1.

**Table 1:** Baseline characteristics of the participants with different depressive symptom trajectories (N=4768).

Variables			Overall		Depressive symptom trajectories									*P* value	
					Type 1		Type 2		Type 3		Type 4				
Total, n (%)			4768 (100)		3656 (76.68)		503 (10.55)		467 (9.79)		142 (2.98)		—^a^		
Age (y), mean (SD)			56.42 (7.87)		57.11 (7.96)		56.41 (7.92)		56.25 (7.96)		56.48 (7.36)		—		
**Age groups (y), n (%)**															.48
	45-54	2069 (43.38)		1595 (43.63)		225 (44.73)		194 (41.54)		54 (38.03)					
	55-64	1938 (40.64)		1477 (40.40)		194 (38.57)		206 (44.11)		61 (42.96)					
	≥65	762 (15.98)		584 (15.97)		84 (16.7)		67 (14.35)		27 (19.01)					
**Sex, n (%)**															<.001
	Male	2574 (53.97)		2098 (57.39)		220 (43.7)		211 (45.18)		45 (31.69)					
	Female	2195 (46.03)		1558 (42.61)		283 (56.2)		256 (54.82)		97 (68.31)					
**Education, n (%)**															<.001
	Below high school	3992 (83.71)		2982 (81.56)		464 (92.25)		412 (88.22)		134 (94.37)					
	High school or above	776 (16.27)		674 (18.44)		39 (7.75)		55 (11.78)		7 (4.93)					
**Area of residence, n (%)**															<.001
	Urban	1087 (22.79)		922 (25.22)		66 (13.12)		79 (16.92)		19 (13.38)					
	Rural	3680 (77.17)		2732 (74.73)		437 (86.88)		388 (83.08)		123 (86.62)					
**Marital status, n (%)**															<.001
	Married with spouse present	4279 (89.73)		3296 (90.15)		450 (89.76)		419 (89.72)		113 (79.58)					
	Married with spouse away	172 (3.61)		129 (3.53)		22 (4.37)		16 (3.43)		5 (3.52)					
	Separated, divorced, or widowed	297 (6.23)		223 (6.1)		26 (5.17)		28 (6)		20 (14.08)					
	Never married	21 (0.44)		8 (0.22)		5 (0.99)		4 (0.86)		4 (2.82)					
**Household income groups, n (%)**															.006
	Quintile 1 (lowest)	948 (19.88)		684 (18.71)		117 (23.26)		109 (23.34)		38 (26.76)					
	Quintile 2	948 (19.89)		714 (19.53)		111 (22.07)		93 (19.91)		30 (21.13)					
	Quintile 3	946 (19.84)		720 (19.69)		96 (19.06)		102 (21.84)		28 (19.72)					
	Quintile 4	944 (19.82)		744 (20.35)		86 (17.1)		89 (19.06)		25 (17.61)					
	Quintile 5 (highest)	946 (19.84)		770 (21.06)		87 (17.3)		69 (14.78)		20 (14.08)					
**Smoking, n (%)**															.008
	Never	2776 (58.21)		2063 (56.43)		327 (65.01)		291 (62.31)		94 (66.20)					
	Current	1598 (33.51)		1275 (34.87)		140 (27.83)		142 (30.41)		41 (28.87)					
	Past	394 (8.26)		317 (8.67)		36 (7.16)		34 (7.28)		7 (4.93)					
**Drinking, n (%)**															<.001
	Never	2659 (55.76)		1956 (53.50)		317 (63.02)		288 (61.67)		97 (68.31)					
	Current	1787 (37.47)		1454 (39.77)		153 (30.42)		147 (31.48)		33 (23.24)					
	Past	323 (6.77)		2446 (6.73)		33 (6.56)		32 (6.85)		12 (8.45)					
**BMI groups (kg/m^2^), n (%)**															.47
	<18.5	163 (3.42)		121 (3.31)		15 (2.98)		18 (3.85)		8 (5.63)					
	18.5-23.9	2079 (43.59)		1586 (43.38)		226 (44.93)		212 (45.40)		55 (38.73)					
	24-27.9	1350 (28.31)		1032 (28.23)		141 (28.03)		130 (27.84)		47 (33.1)					
	≥28.0	619 (12.98)		467 (12.77)		74 (14.71)		59 (12.63)		19 (13.38)					
	Missing	558 (11.7)		450 (12.31)		47 (9.34)		48 (10.28)		13 (9.15)					
**Hypertension, n (%)**															.02
	Yes	1063 (22.29)		801 (21.91)		118 (23.46)		110 (23.55)		34 (23.94)					
	No	3345 (70.14)		2599 (71.09)		340 (67.59)		317 (67.88)		88 (61.97)					
**Diabetes, n (%)**															.33
	Yes	243 (5.10)		177 (4.84)		27 (5.37)		33 (7.07)		6 (4.23)					
	No	4483 (94.00)		3448 (94.31)		472 (93.84)		430 (92.08)		133 (93.66)					
**Cardiovascular disease, n (%)**															.04
	Yes	469 (9.83)		373 (10.2)		37 (7.36)		40 (8.57)		19 (13.38)					
	No	4276 (89.66)		3269 (89.41)		460 (91.45)		424 (90.76)		123 (86.62)					
**Hearing loss, n (%)**															<.001
	Yes	465 (9.75)		333 (9.11)		59 (11.73)		45 (9.64)		28 (19.72)					
	No	4303 (90.25)		3323 (90.89)		444 (88.27)		422 (90.36)		114 (80.28)					

^a^Not available.

### Depressive Symptom Trajectories

In trajectory analysis, we set the number of potential trajectory types to range from 1 to 6 for LGMM modeling, and the performance metrics illustrating the model fitting status of the 6 trajectory models are presented in Table 2. With the increase in the number of trajectory types in LGMM models, the values of the Akaike information criterion, Bayesian information criterion, and sample size–adjusted Bayesian information criterion decreased gradually. Considering the simplicity, accuracy, and practical significance of the developed LGMM models, the model with 4 trajectory types was finally selected as the best fit.

**Figure 2:**
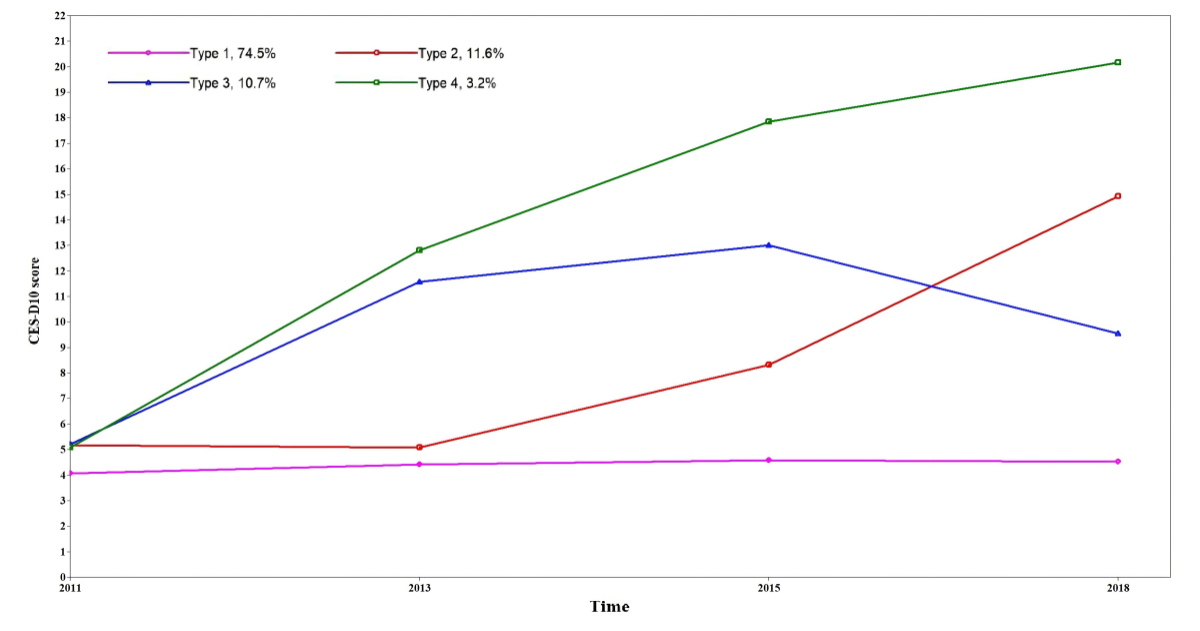
Depressive symptom trajectories for each latent category. CES-D10: 10-item Center for Epidemiologic Studies Depression.

The depressive symptom trajectories of the 4 types (types 1, 2, 3, and 4) are visually displayed in Figure 2. According to the characteristics of each depressive symptom trajectory, we named the 4 trajectories as type 1: “stable low symptoms” (having a steady low CES-D10 score, 74.5%); type 2: “slowly progressing symptoms” (gradually changing from a low to high CES-D10 score, 11.3%); type 3: “relieved symptoms after progression” (with a CES-D10 score growing first and then decreasing, 10.6%); and type 4: “rapidly progressing symptoms” (having a continuously and rapidly increasing CES-D10 score, 3.2%).

The average posterior probability of the most likely latent class membership for participants in each trajectory was calculated for the 4 trajectories, and the results are shown in Table 2. We observed that the average posterior probabilities (diagonal values) were all reasonably high (ranging from 0.779 to 0.939), indicating that the selected trajectory model was acceptable.

**Table 2:** Model fitting statistics of trajectory analysis.

Model fitting statistics			Number of trajectory types										
			1		2		3		4		5		6
AIC^a^			107,515.41		106,781.01		106,390.71		106,168.70		106,063.23		105,926.99
BIC^b^			107,599.52		106,891.00		106,526.58		106,330.45		106,250.86		106,140.49
Entropy			—^c^		0.82		0.85		0.82		0.77		0.78
LMR-LRT^d^, *P* value			—		<.001		<.001		<.001		.26		.33
BLRT^e^, *P* value			—		<.001		<.001		<.001		<.001		<.001
**Class proportion (%)**													
	Type 1	100		85.66		82.48		74.51		48.71		45.84	
	Type 2	—		14.34		8.77		11.60		5.96		4.36	
	Type 3	—		—		8.75		10.74		35.10		3.62	
	Type 4	—		—		—		3.16		3.25		35.74	
	Type 5	—		—		—		—		7.00		5.87	
	Type 6	—		—		—		—		—		4.57	
**Average posterior probabilities**													
	Type 1	1		0.961		0.802		0.789		0.887		0.890	
	Type 2	—		0.865		0.821		0.779		0.752		0.743	
	Type 3	—		—		0.957		0.843		0.843		0.724	
	Type 4	—		—		—		0.938		0.789		0.838	
	Type 5	—		—		—		—		0.780		0.770	
	Type 6	—		—		—		—		—		0.753	

^a^AIC: Akaike information criterion.

^b^BIC: Bayesian information criterion.

^c^Not available.

^d^LMR-LRT: Lo-Mendell-Rubin Likelihood Ratio Test.

^e^BLRT: Bootstrap Likelihood Ratio Test.

### Associations Between Hearing Loss and Depressive Symptom Trajectories

The results of logistic regression analyses for the associations between hearing loss and depressive symptom trajectories are presented in Table 3. Participants with hearing loss had a higher risk of having a “rapidly progressing symptoms” trajectory than the risk of the other 3 trajectories. Then, we conducted a subgroup analysis stratified by gender. Results showed that in female participants with hearing loss, they had a higher risk of having a “rapidly progressing symptoms” trajectory than “stable low symptoms” or “relieved symptoms after progression” trajectories, but the risks of having “rapidly progressing symptoms” and “slowly progressing symptoms” showed no difference. In male participants with hearing loss, the risk of having a “rapidly progressing symptoms” trajectory was still higher with statistical significance (*P*=.02) than the other 3 depressive symptom trajectories, which suggested that hearing loss may have a more substantial impact on depressive symptoms in male individuals.

**Table 3:** Odds ratios and 95% CIs for different depressive symptom trajectories associated with baseline hearing loss.

Predictors and depressive symptom trajectories							
	Type 1	Type 2		Type 3		Type 4	
		OR (95% CI)	*P* value	OR (95% CI)	*P* value	OR (95% CI)	*P* value
**Hearing loss in men and women (N=4768)^a^**							
	Ref^b^	1.33 (0.99-1.80)	.06	1.07 (0.76-1.49)	.70	2.41 (1.54-3.76)	<.001
	—^c^	Ref	—	0.80 (0.52-1.21)	.29	1.92 (1.14-3.23)	.01
	—	—	—	Ref	—	2.49 (1.45-4.27)	<.001
**Hearing loss in men (n=2574)^d^**							
	Ref	1.20 (0.79-1.82)	.40	1.12 (0.72-1.75)	.60	2.67 (1.29-5.50)	.008
	—	Ref	—	0.93 (0.52-1.68)	.82	2.86 (1.17-6.98)	.02
	—	—	—	Ref	—	2.76 (1.16-6.56)	.02
**Hearing loss in women (n=2194)^d^**							
	Ref	1.51 (0.98-2.34)	.06	0.98 (0.59-1.64)	.94	2.38 (1.34-4.23)	.003
	—	Ref	—	0.71 (0.38-1.30)	.27	1.90 (0.97-3.73)	.06
	—	—	—	Ref	—	2.58 (1.25-5.34)	.01

^a^Adjusted for age, sex, education level, area of residence, marital status, household income, smoking status, drinking status, BMI, hypertension, diabetes, and cardiovascular disease.

^b^Ref: reference.

^c^Not available.

^d^Adjusted for age, education level, area of residence, marital status, household income, smoking status, drinking status, BMI, hypertension, diabetes, and cardiovascular disease.

### The Role of Social Engagement

Logistic regression analyses revealed a significant association between hearing loss and reduced social engagement after adjusting for confounding variables (OR 0.79, 95% CI 0.62-0.99). Concurrently, the risk of exhibiting a rapidly progressing symptoms trajectory (vs stable low symptoms) after adjustment for social engagement (OR 2.19, 95% CI 1.30-3.70) was lower than before (OR 2.49, 95% CI 1.45-4.27). However, social engagement did not demonstrate a significant mediating effect in the relationship between hearing loss and depressive symptom trajectories. This suggests that the influence of hearing loss on depressive symptom progression may involve other factors, such as neurophysiological mechanisms. The conclusion remained consistent in gender-stratified analysis.

## Discussion

### Principal Findings

On the basis of a nationally representative dataset of middle-aged and older Chinese adults without depressive symptoms at baseline (2011), we used the LGMM approach to perform a depressive symptom trajectory analysis in this study, and identified 4 trajectory types: type 1, “stable low symptoms”; type 2, “slowly progressing symptoms”; type 3, “relieved symptoms after progression”; and type 4, “rapidly progressing symptoms.” Furthermore, we examined the impact of hearing loss on the depressive symptom trajectories. We found that hearing loss was significantly associated with the “rapidly progressing symptoms” trajectory, and the impact was more substantial in males. The association between hearing loss and rapidly progressing depressive symptoms was attenuated after adjustment for social engagement, but no significant evidence was found to support a mediating role of social isolation in the association between hearing loss and depressive symptom trajectories.

### Comparison With Prior Work

The findings regarding the significant association between hearing loss and the rapid progression of depressive symptoms in this study are consistent with the conclusions of some existing studies. For instance, Simning et al [42] examined the relationship between hearing loss and depression in a study encompassing a US population aged 65 years and older, and found that hearing loss was significantly linked not only to the onset of depressive symptoms within 1 year but also to the risk of persistent depressive symptoms. Kiely et al [43] found that individuals with hearing loss exhibited more severe depressive symptoms compared to those without sensory impairments in their examination of 16 years of longitudinal data gathered by the Australian Longitudinal Study of Aging. Nevertheless, a few other studies have different findings. For example, Cosh et al [27] found a significant association between hearing loss and depression in cross-sectional data at baseline in a study involving 2890 Norwegians aged 60 years and older but did not observe a longitudinal association in the 6-year follow-up. This inconsistency in research findings may be attributed to variations in the criteria used to define hearing loss and depressive symptoms. In addition, we observed that there was a gender difference in the impact of hearing loss on depressive symptom trajectories. While the risk of having a “rapidly progressing symptoms” trajectory was significantly higher than the risk of the 3 other depressive symptom trajectories in male participants with hearing loss, the risk of having a “rapidly progressing symptoms” or “slowly progressing symptoms” trajectory did not differ in female participants with hearing loss. This means that hearing loss may impact both the incidence and progression speed of depressive symptoms in males, but may not affect the depressive symptom progression speed in female individuals. Previous studies also found that the association between hearing loss and the risk of depressive symptoms was more significant in male individuals. These studies suggest that communication barriers resulting from hearing impairment, along with consequent social isolation, may exert a more pronounced effect in male individuals by exacerbating their already restricted social interactions [44-46].

It is generally believed that the mechanism behind this association operates through social isolation. Hearing problems lead to communication difficulties, making social interactions challenging, causing people to withdraw from social activities, and consequently generating feelings of loneliness and depression [47]. Studies indicate that after adjusting for social contact, the association between hearing loss and depressive symptoms weakened and became nonsignificant [43]. Research by Huang et al [30] showed that in mediation effect analysis, the proportion of the association mediated by social isolation was 9%.

However, in this study, after adjusting for social engagement, the association between hearing loss and depressive symptom trajectories remained significant, but no significant mediating effect was found. A study by Keidser and Seeto [47] demonstrated an independent association between hearing loss and depressive episodes, with social isolation having minimal influence on this association, which aligns with our findings. This outcome may be due to the fact that the social participation variable in this study relates to the quantity rather than the satisfaction of social interactions, and hearing-impaired individuals may not have withdrawn from social activities but rather become unable to engage in effective communication [48]. Additionally, our study population primarily consisted of middle-aged and older adults who may have adapted to changes in hearing conditions, with their communication skills having been changed or improved. Hawthorne [49] also found that the proportion of social isolation was higher among young people (ages 15-30 years) than among older adults (>60 years). It may be inferred that factors, such as social engagement, are more likely to mediate the association between hearing problems and depression in young and middle-aged adults compared to older adults. Furthermore, this study did not exclude hearing aid users. Older adults may use hearing aids to improve their hearing conditions, potentially mitigating the negative impacts of hearing loss [30].

Social engagement cannot fully explain the association between hearing loss and depressive symptoms, and potential neuropsychological mechanisms may exist. Neuroimaging studies have revealed similar alterations in patients with hearing loss and individuals with depression [50,51]. Additionally, research indicates that responses to emotional sounds in the amygdala and parahippocampal gyrus are diminished among hearing-impaired individuals [31]. However, the precise neuropsychological mechanisms remain unclear and warrant further investigation.

Our study demonstrates that hearing loss constitutes a risk factor for the rapid progression of depressive symptoms, warranting increased attention from clinicians and psychologists. It is imperative to enhance monitoring of mental health status among middle-aged and older adults with hearing impairment while proactively implementing auditory rehabilitation interventions, such as hearing aids, cochlear implants, or virtual reality cognitive–based interventions to mitigate the impact of hearing loss on patient well-being [52].

### Strengths and Limitations

A strength of this study was a nationally representative, large-scale dataset with a high response rate and a rigorous quality control process, ensuring the reliability of the findings generated from the dataset. Furthermore, the 8-year follow-up period (2011-2018) in this study was sufficient to identify distinct and clinically meaningful trajectories of depressive symptoms, as many studies on depression trajectories have used similar follow-up periods [53-55]. The trajectory analysis showed 4 distinct categories, indicating that an 8-year follow-up period can provide a certain degree of generalization of the development trajectory of patients’ depressive symptoms.

However, this study still has its limitations. First, the definition of hearing loss in this study was based solely on participants’ self-reports, rather than results derived from clinical measurements [54]. The clinical hearing test was conducted without assistance, whereas self-reported hearing may reflect the hearing experience with the help of a hearing aid in daily life. This study did not exclude hearing aid users, and older adults may use hearing aids to improve their hearing conditions, potentially mitigating the negative impacts of hearing loss. Nevertheless, the objective measurement of hearing correlates well with the subjective sensory assessment [56]. Moreover, it has been suggested that self-reported hearing loss may better reflect the extent to which patients’ daily lives are affected and may be more valuable when exploring the relationship between hearing loss and mental health status [20,57,58]. Second, some factors, such as psychiatric and medication history, were not available in the CHARLS dataset, and this may impact the analysis results. Third, due to the use of a preexisting national database, this study was limited by the inability to incorporate qualitative data, such as in-depth interviews, and insights into potential mechanisms were then supplemented through a literature review and mediation analysis of social engagement measures. Fourth, considering individuals who already have depression symptoms at baseline are likely to have received interventions or developed adaptive mechanisms, which could substantially alter the natural progression of depressive symptoms and confound the estimated effect of hearing loss, we excluded participants with baseline depression symptoms. However, this exclusion may limit the generalization of our findings to all depression trajectory types, and future studies incorporating participants across all baseline depression statuses would be valuable to capture a fuller spectrum of symptom trajectories.

### Conclusions

To summarize, this study was the first to analyze the trajectories of depressive symptoms in Chinese middle-aged and older adults without depressive symptoms at baseline, and further explored the association between hearing loss and depressive symptom trajectories. Findings of this study showed that the depressive symptom trajectories in Chinese middle-aged and older adults could be categorized into 4 types, including “stable low symptoms,” “slowly progressing symptoms,” “relieved symptoms after progression,” and “rapidly progressing symptoms.” Hearing loss was significantly associated with the “rapidly progressing symptoms” trajectory among the study population, and the impact was stronger for males. It suggests that more attention should be paid to the mental status of Chinese middle-aged and older adults with hearing loss, and if possible, appropriate interventions should be taken to prevent the onset of depressive symptoms.

## Appendix

Multimedia Appendix 1 The 10-item Center for Epidemiologic Studies Depression (CES-D10) scale.

Multimedia Appendix 2 Survey questions related to hearing in the China Health and Retirement Longitudinal Study.

## Abbreviations

CES-D10: 10-item Center for Epidemiologic Studies Depression
CHARLS: China Health and Retirement Longitudinal Study
CVD: cardiovascular disease
LGMM: latent growth mixture modeling

## Data Availability

The data that support the findings of this study are available from the China Health and Retirement Longitudinal Study [59].
