# Supplementary material for: Associations Between Hearing Loss and Depressive Symptom Trajectories in Middle-Aged and Older People in China: Retrospective Analysis
Source: JMIR Aging. 2025 Nov 24;8:e75545. doi: 10.2196/75545 (PMC12686856; doi:10.2196/75545)
Supplement: Multimedia Appendix 1 [file aging_v8i1e75545_app1.docx]

**Supplementary Table 1:** The CES-D10 (Center for Epidemiologic Studies Depression Scale)

| The 10 items below refer to how you have felt and behaved during the last week. Choose the appropriate response.   1. I was bothered by things that don't usually bother me. 2. I had trouble keeping my mind on what I was doing. 3. I felt depressed. 4. I felt everything I did was an effort. 5. I felt hopeful about the future. 6. I felt fearful. 7. My sleep was restless. 8. I was happy. 9. I felt lonely. 10. I could not get "going." |
| --- |
