# Supplementary material for: Associations Between Hearing Loss and Depressive Symptom Trajectories in Middle-Aged and Older People in China: Retrospective Analysis
Source: JMIR Aging. 2025 Nov 24;8:e75545. doi: 10.2196/75545 (PMC12686856; doi:10.2196/75545)
Supplement: Multimedia Appendix 2 [file aging_v8i1e75545_app2.docx]

**Supplementary Table 2:** Survey questions related to hearing in CHARLS.

| 1. Do you have a hearing disability?  (1) Yes  (2) No  2. Do you usually wear a hearing aid?  (1) Yes  (2) No  3. Is your hearing excellent, very good, good, fair, or poor? (How is your hearing with  a hearing aid if you normally use it? How is your hearing without a hearing aid if  you normally don’t use it?)  (1) Excellent  (2) Very good  (3) Good  (4) Fair  (5) Poor |
| --- |
